# High resolution flat-panel CT arthrography *vs*. MR arthrography of artificially created osteochondral defects in *ex vivo* upper ankle joints

**DOI:** 10.1371/journal.pone.0255616

**Published:** 2021-08-10

**Authors:** Gesa H. Pöhler, Lena Sonnow, Sarah Ettinger, Alexandra Rahn, Filip Klimes, Christoph Becher, Christian von Falck, Frank K. Wacker, Christian Plaass

**Affiliations:** 1 Department of Diagnostic and Interventional Radiology, Hannover Medical School, Hannover, Germany; 2 Department of Orthopedic Surgery, Hannover Medical School at Diakovere Annastift, Hannover, Germany; 3 International Centrum for Hip, Knee and Foot Surgery, ATOS Clinic Heidelberg, Heidelberg, Germany; Johns Hopkins School of Medicine, UNITED STATES

## Abstract

**Purpose:**

High resolution flat-panel computed tomography arthrography (FPCT-A) and magnetic resonance arthrography (MR-A) are well suited to evaluate osteochondral lesions. The current study compares the performance of FPCT-A versus MR-A in an experimental setting.

**Methods:**

Fourteen cadaveric ankles were prepared with artificial osteochondral defects of various sizes in four separate talar locations. After intra-articular contrast injection, FPCT-A and 3-T MR-A were acquired. Each defect was then filled with synthetic pallets. The resulting cast was used as reference. Two independent radiologists measured the dimensions of all defects with FPCT-A and MR-A. Intra-class correlation coefficients (ICC) were calculated. Data were compared using t-tests and Bland-Altman plots.

**Results:**

The correlation for FPCT-A and cast was higher compared to MR-A and cast (ICC 0.876 *vs*. 0.799 for surface [length x width]; ICC 0.887 *vs*. 0.866 for depth, p<0.001). Mean differences between FPCT-A and cast measurements were -1.1 mm for length (p<0.001), -0.7 mm for width (p<0.001) and -0.4 mm for depth (p = 0.023). By MR-A, there were no significant differences for length and width compared to cast (p>0.05). Depth measurements were significantly smaller by MR-A (mean difference -1.1 mm, p<0.001). There was no bias between the different modalities.

**Conclusions:**

*Ex vivo* FPCT-A and MR-A both deliver high diagnostic accuracy for the evaluation of osteochondral defects. FPCT-A was slightly more accurate than MR-A, which was most significant when measuring lesion depth.

## Introduction

At the ankle, osteochondral lesions are often traumatic and painful [1, 2]. The lesions typically involve the articular cartilage and subchondral bone, mostly the talus. Surgery is required in many cases. The optimal form of surgical treatment depends on defect characteristics, such as size and location [3–7]. Further, defect size correlates with patient outcome [8]. Therefore, it is imperative to be able to precisely determine the extent of any osteochondral defects at the ankle [9].

Routine magnetic resonance imaging (MRI) without intravenous or intra-articular contrast agent is an option to evaluate the cartilage at the ankle [10–12]. Standard MRI, however, may be limited and overestimate the size of a lesion due to bone edema [13]. Direct magnetic resonance arthrography (MR-A) could be considered for further work up. MR-A better detects chondral defects, especially in patients with lesions of the upper ankle cartilage [14].

Improved dose reduction management and technology in multidetector computed tomography (MDCT) have enabled MDCT to also detect thin cartilaginous lesions at the ankle [15]. Recently, flat panel computed tomography (FPCT) was established as an imaging modality with superior spatial resolution including submillimeter isotropic voxel sizes compared to MDCT [16, 17]. The radiation dose is similar for FPCT and MDCT [17]. FPCT further presents reduced metal and beam-hardening artifacts [18]. Finally, FPCT is widely available as a part of most angiography suites. There, fluoroscopic guided contrast injection and cross-sectional arthrography can be performed in a single session without need for patient transfer [19, 20].

Despite the plethora of various imaging techniques, a definitive diagnosis of an osteochondral defect can be challenging [18, 21]. Controversy exists regarding the imaging modality of choice [2, 22–24]. Thus, the current study investigates and compares the diagnostic performance of high resolution FPCT-A versus MR-A in an experimental setup.

## Materials and methods

### Cadaver preparation

The study was waived by the local Institutional Review Board (Medical School Hannover). Fourteen fresh-frozen cadaveric feet with lower legs were recruited for the study (Science Care). Any prior surgical treatment resulted in exclusion. The upper ankle joint was opened by a mini-arthrotomy and inspected for already existing osteochondral lesions. Only cadavers without any defects were used. Artificial defects of different sizes and depths were then created with different surgical curettes. The lesions were established in four predefined locations at the talus: anteromedial, anterolateral, posteromedial and posterolateral. Collateral soft tissue damage was avoided during the procedure. Then, the joint capsule and skin were closed with surgical sutures.

### Contrast application and image acquisition

The cadavers were positioned with the lateral malleolus facing upwards. The injection was performed under fluoroscopic guidance using a 20G needle. The contrast consisted of a 1:1 mixture of an iodinated agent (Iomeprol 300 mg/ml, Imeron 300, Bracco Imaging) and a pre-diluted gadolinium-based solution (Gadolinium-DTPA 2 mmol/l, Dotarem 0,5 mmol/ml, Guerbet). 5–10 ml contrast mixture was injected into each joint. Then, the ankle was passively moved to ensure homogeneous distribution of the contrast agent within the joint.

Following the injection, FPCT-A was acquired with the same angiographic system (ARTIS pheno AXH 1964, Siemens Healthineers). Tube voltage was 70 kV, tube current was 400–410 mA. The average dose-area product was 685 μGym^2^. Images were reconstructed using the smallest possible volume-of-interest per ankle and the “normal” bone reconstruction kernel. The resulting voxel size was in the range of 0.11–0.12 mm.

After transfer to the MRI suite, MR images were acquired using a 3T MR scanner (MAGNETOM Skyra, Siemens Healthineers) and a dedicated 20-channel phase array head coil. As imaging protocol, the protocol already established for clinical application was used comprised of the following: T1-weighted (w) Turbo Spin Echo (TSE) sequences with spectral fat suppression (FS) in three planes; sagittal T1w TSE sequence without FS; a proton density weighted (PDw) coronal sequence; an isotropic 3D PD space sequence. The detailed scan parameters are summarized in Table 1.

**Table 1:** MR scan parameters.

MR-A	T1w TSE	T1w TSE	T1w TSE	PDw TSE	T1w TSE	3DTSE SPACE
Parameters	FS		FS	FS	FS	FS
Orientation	transverse	sagittal	sagittal	coronal	coronal	sagittal
						3D block
Repetition time (ms)	601	956	1010	2660	721	1000
Echo time (ms)	13	13	13	33	13	43
Receiver bandwidth	257	257	257	200	257	460
(Hertz/pixel)						
Flip angle (degree)	150	150	150	150	150	variable
Field-of-view (mm^2^)	130 x 110	130 x 130	130 x 130	130 x 102	130 x 130	180 x 170
Matrix	512 x 367	512 x 512	512 x 512	512 x 512	512 x 512	320 x 285
Slice thickness / gap (mm)	2.0/0	2.0/0	2.0/0	2.0/0	2.0/0	0.6/0
Pixel dimensions (mm^2^)	0.3 x 0.3	0.3 x 0.3	0.3 x 0.3	0.3 x 0.3	0.3 x 0.3	0.6 x 0.6
Number of Slices	30	32	30	40	33	112
Phase encoding direction	anterior to posterior	head to foot	head to foot	right to left	right to left	head to foot
Acquisition time (min:sec)	4:02	4:22	4:40	3:08	3:45	7:26

Magnetic Resonance Arthrography (MR-A) scanning parameters

TSE = turbo spin echo; FS = fat saturated; PDw = proton density weighted; space = sampling perfection with application optimized contrasts using different flip angle evolution

### Preparation of casting specimens

After arthrography, the cadaveric ankles were disarticulated and the talar surface was carefully prepared. In order to obtain a reference size for each lesion, the defects were filled with synthetic pallets (Friendly plastic, AMACO). This created a reference cast for each defect. The casts were labeled according to the defect location and a high-resolution FPCT-scan was acquired for measurements of the casts.

### Image assessment and measurements

**Fig 1:**
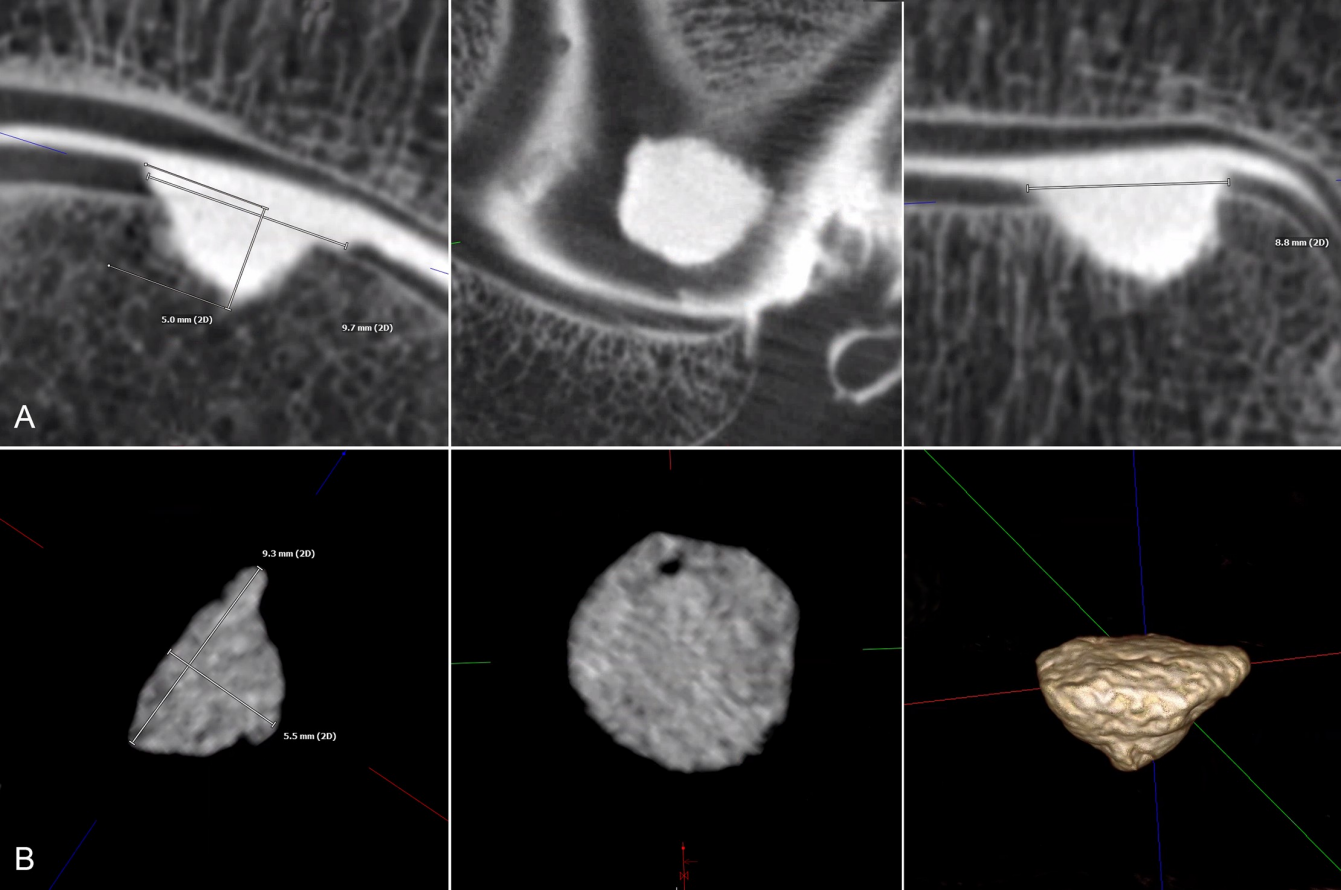
Osteochondral defect measurement. Example of measurements in flat panel computed tomography arthrography (FPCT-A) with a multiplane reconstruction of the same defect in three planes (A) and the corresponding cast specimen (B) in two planes and a 3D rendering mode.

Two independent radiologists with more than 5 years in practice evaluated all FPCT-A and MR-A images. The radiologists were blinded to the identifying information of the images. Images were evaluated on a client-server based picture archiving and communication system (PACS) workstation (Visage 7.1, Visage Imaging). The raters used the 3D functionality of the software including multiplanar reformations, thin slab averaging and fusion/overlay mode. Regarding the MR-images, the radiologists evaluated defect configuration on all MR sequences and used the 3D SPACE sequence in the multiplanar mode to measure the dimensions of the defects. Defect depth was measured as illustrated in Fig 1A. Defect length was defined as the largest extent at the cartilage level, defect width was measured at the largest extent of a perpendicular line to the defect’s length (Fig 1A). Defect length and width were multiplied and defined as the defect’s surface area (mm^2^) and used for calculation of correlation coefficients and Bland-Altman plots. Measurement of the casting specimens included visualization in multiplane reconstruction and 3D mode with a clear depiction of defect shape. Then, the cast’s length, width and depth were identified and measured in three planes. The results were calculated similarly to the osteochondral defect measurements (Fig 1B).

### Statistical analysis

SPSS Statistics 27.0.0.0 (IBM Corp) was used for the statistical analysis. Tests on normal distribution (Kolmogorov-Smirnow) for both readers and all FPCT-, MR-A and cast specimen measurements were performed. The defect measurements of reader 1 were compared to reader 2 using a t-test. The inter-reader correlation was calculated using intra-class correlation coefficient (ICC) with associated p value. The means of corresponding measurements of the two radiologists were calculated and used for comparison/correlation of FPCT-A, MR-A and cast.

Reliability between both imaging modalities and casting specimens were calculated using ICCs with a 95% confidence interval in a two-way, mixed, consistency model [25]. The ICC values were interpreted as follows: values < 0.4 reflected poor correlation, 0.4–0.59 intermediate, 0.60–0.74 good, and 0.75–1.00 very good correlation [26]. Test on normal distribution (Kolmogorov-Smirnow) showed normal distribution for all FPCT-, MR-A and cast specimen measurements. A t-test for paired samples was used for comparison of the measurements of FPCT-A *vs*. cast, MR-A *vs*. cast and FPCT-A *vs*. MR-A. A p-value < 0.05 was considered significant. Bland-Altman plots were calculated. The upper and lower limit of agreement range was calculated according to: mean difference +/- standard deviation x 1.96.

## Results

All four artificially created defects in each ankle joint were detected by both the FPCT- and MR-images. The defect measurements of reader 1 compared to reader 2 showed no significant difference (p>0.05). Indeed, the inter-reader correlation between reader 1 and 2 showed “very good” agreement (ICC >0.9, 95% CI 0.984–0.989, p<0.001). The mean values of corresponding measurements of the two radiologists are presented in Tables 2 and 3.

**Table 2:** Defect measurements cadavers 1–7.

Cadaver No.	Talar defect location	Surface in mm^2^			Depth in mm		
		mean of both readers (SD)			mean of both readers (SD)		
		FPCT-A	MR-A	Cast	FPCT-A	MR-A	Cast
1	AM	28.8 (0.0)	41.4 (7.8)	32.9 (3.1)	4.9 (0.1)	4.8 (0.0)	4.6 (0.1)
	AL	47.2 (1.4)	162.5 (46.32)	34.4 (11.9)	3.6 (0.0)	3.5 (0.0)	2.1 (0.1)
	PM	45.7 (1.5)	50.7 (1.6)	52.0 (7.9)	14.3 (0.3)	14.0 (0.0)	9.6 (0.0)
	PL	38.9 (0.0)	41.0 (2.4)	55.4 (3.3)	5.3 (0.0)	4.0 (0.0)	3.9 (0.1)
2	AM	76.8 (0.0)	93.3 (0.4)	75.8 (21.4)	6.4 (0.0)	5.4 (0.1)	8.2 (0.1)
	AL	37.5 (0.0)	43.4 (0.9)	36.6 (9.5)	5.7 (0.0)	4.7 (0.1)	5.8 (0.1)
	PM	42.4 (0.0)	44.8 (0.4)	42.3 (6.4)	6.8 (0.0)	4.9 (0.0)	5.0 (1.8)
	PL	65.1 (0.0)	68.9 (2.3)	65.98(7.67)	6.6 (0.0)	4.8 (0.1)	6.9 (2.7)
3	AM	73.9 (0.0)	92.4 (0.9)	70.7 (15.8)	4.0 (0.0)	4.0 (0.1)	4.9 (1.1)
	AL	42.6 (0.0)	51.2 (4.0)	77.04 (0.76)	1.6 (0.0)	1.5 (0.0)	3.2 (0.8)
	PM	65.5 (0.0)	69.7 (1.2)	73.6 (11.2)	5.5 (0.0)	4.0 (0.1)	5.3 (0.8)
	PL	41.6 (0.0)	41.1 (3.9)	48.8 (13.7)	2.7 (0.0)	2.5 (0.0)	2.7 (0.4)
4	AM	34.8 (0.0)	48.6 (0.5)	92.3 (44.7)	1.4 (0.1)	1.3 (0.1)	2.0 (1.1)
	AL	52.9 (0.5)	66.0 (1.7)	76.0 (6.0)	4.2 (0.1)	2.6 (0.1)	4.2 (0.6)
	PM	72.7 (1.4)	78.2 (1.3)	90.0 (7.0)	2.4 (0.3)	2.1 (0.1)	6.4 (0.4)
	PL	36.3 (11.0)	51.6 (0.0)	55.8 (9.0)	4.0 (0.9)	2.2 (0.0)	4.5 (0.5)
5	AM	97.1 (0.5)	106.5 (2.9)	138.8 (9.4)	6.1 (0.0)	3.2 (0.1)	4.7 (0.4)
	AL	38.7 (3.8)	48.2 (0.4)	75.5 (9.2)	4.5 (0.1)	3.8 (0.3)	4.2 (0.6)
	PM	93.5 (0.7)	98.2 (0.7)	89.3 (6.3)	5.4 (0.1)	5.4 (0.1)	6.8 (0.8)
	PL	36.0 (0.0)	64.3 (4.7)	63.8 (2.0)	5.1 (0.1)	3.3 (0.1)	4.2 (0.6)
6	AM	92.9 (8.5)	122.3 (0.7)	114.2 (24.2)	5.9 (0.2)	5.4 (0.1)	5.6 (0.1)
	AL	61.8 (0.5)	87.7 (0.7)	94.9 (13.7)	1.6 (0.0)	1.6 (0.1)	2.9 (0.4)
	PM	79.6 (1.97)	139.1 (14.0)	92.4 (26.5)	5.0 (0.1)	4.8 (0.0)	6.2 (0.1)
	PL	42.6 (2.3)	57.5 (0.1)	62.8 (2.6)	4.9 (0.1)	3.8 (0.1)	7.2 (0.1)
7	AM	112.7 (2.4)	119.5 (0.0)	89.6 (2.2)	5.7 (0.1)	4.7 (0.1)	6.6 (0.0)
	AL	35.6 (0.1)	66.8 (0.6)	74.6 (0.0)	1.2 (0.1)	1.1 (0.1)	2.0 (0.5)
	PM	34.8 (1.7)	66.0 (0.7)	44.7 (4.7)	1.3 (0.1)	2.4 (0.1)	2.6 (0.0)
	PL	74.1 (0.8)	119.9 (1.5)	71.9 (10.9)	3.2 (0.1)	4.4 (0.2)	4.1 (0.2)

Mean values of both readers for each localization in cadavers1-7 for surface and depth measurements in FPCT-A, MR-A and cast respectively. Standard deviation (SD) in parantheses. *AM = anteromedial*, *AL = anterolateral*, *PM = posteromedial and PL = posterolateral*.

**Table 3:** Defect measurements cadavers 8–14.

Cadaver No.	Talar defect location	Surface in mm^2^			Depth in mm		
		mean of both readers (SD)			mean of both readers (SD)		
		FPCT-A	MR-A	Cast	FPCT-A	MR-A	Cast
8	AM	83.0 (0.0)	97.1 (2.7)	84.2 (17.5)	1.5 (0.0)	1.6 (0.1)	2.9 (0.3)
	AL	81.8 (1.1)	119.8 (3.1)	104.4 (6.6)	3.4 (0.1)	3.1 (0.1)	4.6 (0.4)
	PM	77.9 (0.2)	93.2 (8.8)	100.5 (0.7)	4.5 (0.0)	3.9 (0.2)	5.4 (0.3)
	PL	36.5 (0.0)	36.3 (0.4)	61.0 (2.7)	1.5 (0.1)	1.4 (0.0)	2.2 (0.2)
9	AM	89.9 (0.0)	76.3 (3.6)	87.7 (12.6)	2.9 (0.0)	1.8 (0.0)	3.8 (0.3)
	AL	83.7 (0.0)	109.7 (34.6)	97.6 (8.8)	3.4 (0.0)	2.3 (0.5)	3.3 (0.0)
	PM	97.5 (0.0)	56.0 (3.5)	94.4 (24.0)	5.1 (0.0)	2.2 (0.3)	5.7 (0.8)
	PL	75.6 (0.0)	85.5 (3.5)	108.5 (0.2)	4.2 (0.0)	2.5 (0.0)	5.0 (0.7)
10	AM	77.8 (2.5)	120.1 (20.3)	105.4 (14.7)	4.5 (0.1)	3.3 (0.1)	4.3 (0.4)
	AL	115.5 (6.2)	100.4 (4.94)	162.7 (3.8)	5.4 (0.1)	4.9 (0.0)	6.3 (0.5)
	PM	84.2 (0.7)	93.1 (1.4)	82.3 (6.6)	7.8 (0.2)	6.7 (0.4)	7.4 (0.5)
	PL	82.5 (9.2)	81.4 (5.9)	95.9 (16.9)	2.0 (0.0)	1.8 (0.1)	2.6 (0.1)
11	AM	163.1 (17.5)	275.5 (0.0)	200.2 (24.5)	6.3 (0.1)	5.4 (0.0)	6.2 (0.0)
	AL	120.2 (2.6)	104.2 (36.4)	94.9 (12.5)	1.1 (0.1)	1.4 (0.2)	2.5 (0.4)
	PM	197.6 (3.7)	96.0 (8.5)	127.3 (13.6)	6.4 (0.1)	6.3 (0.1)	7.5 (0.1)
	PL	83.2 (0.1)	93.5 (2.0)	107.3 (5.6)	2.7 (0.2)	2.3 (0.3)	4.5 (0.5)
12	AM	110.3 (1.8)	175.2 (2.7)	156.3 (54.2)	4.8 (0.1)	3.3 (0.1)	4.0 (0.7)
	AL	85.64 (1.3)	86.5 (4.4)	105.6 (16.2)	4.1 (0.1)	4.0 (0.1)	5.5 (0.0)
	PM	77.0 (1.4)	71.8 (2.7)	103.9 (2.8)	1.6 (0.0)	1.7 (0.1)	2.7 (0.1)
	PL	43.5 (0.0)	67.6 (0.7)	74.6 (10.3)	4.7 (0.1)	4.8 (0.1)	4.4 (0.7)
13	AM	99.4 (0.0)	129.5 (2.0)	115.0 (33.6)	3.2 (0.2)	3.4 (0.1)	3.3 (0.1)
	AL	50.1 (1.6)	42.0 (12.8)	109.7 (12.4)	3.7 (0.1)	2.7 (0.1)	3.7 (0.1)
	PM	58.0 (0.5)	71.8 (1.8)	60.9 (5.7)	4.3 (0.4)	3.9 (0.1)	5.5 (0.6)
	PL	52.3 (4.6)	43.4 (0.1)	65.1 (4.5)	4.4 (0.1)	4.3 (0.1)	4.0 (1.5)
14	AM	82.7 (0.0)	88.7 (0.2)	83.5 (12.9)	4.3 (0.0)	2.6 (0.1)	4.1 (0.8)
	AL	111.0 (0.0)	131.8 (3.2)	152.2 (14.6)	5.6 (0.0)	3.7 (0.3)	6.1 (1.6)
	PM	91.0 (0.0)	103.3 (5.0)	103.0 (5.1)	3.3 (0.0)	2.1 (0.1)	3.7 (0.6)
	PL	65.3 (0.0)	71.1 (11.2)	70.5 (0.4)	5.3 (0.0)	4.7 (0.2)	5.3 (0.4)

Mean values of both readers for each localization in cadavers 8–14 for surface and depth measurements in FPCT-a, MR-A and cast respectively. Standard deviation (SD) in parantheses. *AM = anteromedial*, *AL = anterolateral*, *PM = posteromedial and PL = posterolateral*.

### T-test results

The defects were measured significantly smaller compared to the cast using FPCT-A: mean differences were -1.1 mm for length (p<0.001), -0.7 mm for width (p<0.001) and -0.4 mm for depth (p = 0.023). By MR-A, there was no significant difference for length and width compared to the cast, whereas depth was measured significantly smaller in MR-A (mean difference -1.1 mm, p<0.001). Overall, defect width and length were measured significantly smaller by FPCT-A compared to MR-A (p<0.001), whereas depth was measured +0.7 mm larger in FPCT-A (p<0.001). The detailed results are given in Table 4.

**Table 4:** T-test results.

Defect size parameter	Modalities	Mean difference	[95% CI]	t-value	p-value
**Length**	**FPCT-A–cast**	-1.1	[-1.7; -0.5]	-3.8	<0.001
**(mm)**					
	**MR-A–cast**	-0.6	[-1.2; 0.0]	-1.9	0.062
	**FPCT-A–MR-A**	-0.5	[-1.1; 0.0]	-2.0	0.056
**Width**	**FPCT-A–cast**	-0.7	[-1.0; -0.4]	-4.3	<0.001
**(mm)**					
	**MR-A–cast**	0.2	[0.6; 1.1]	1.1	0.289
	**FPCT-A–MR-A**	-0.9	[-1.2; -0.6]	-5.7	<0.001
**Depth**	**FPCT-A–cast**	-0.4	[-0.7; -0.1]	-2.3	0.023
**(mm)**					
	**MR-A–cast**	-1.1	[-1.4; -0.7]	-6.4	<0.001
	**FPCT-A–MR-A**	0.7	[0.5; 0.9]	6.2	<0.001

Results of the paired t-test with mean difference of defect length, width and depth (in mm) between the flat panel computed tomography arthrography (FPCT-A), magnetic resonance arthrography (MR-A) and cast specimens. Data are presented with 95% confidence intervals (CI), t-values and p-values.

### Correlation results

All calculated ICCs between FPCT-A, MR-A and cast showed very good correlation (ICC >0.75, p<0.001). The measurements of the defect surface (length x width) showed a higher correlation for FPCT-A and cast (ICC 0.876, p<0.001) compared to MR-A and cast (ICC 0.799, p<0.001). Similarly, depth measurements correlated slightly better between FPCT-A and cast (0.887, p<0.001) compared to MR-A and cast (0.866, p<0.001). We observed a high correlation of the depth measurements between FPCT-A and MR-A (0.957, p<0.001). The detailed results are given in Table 5.

**Table 5:** Correlation results.

Defect size parameter	Modalities	ICC	95% CI	p-value
**Surface (mm** ^**2**^ **)**	**FPCT-A *vs*. cast**	0.876	[0.788; 0.927]	<0.001
	**MR-A *vs*. cast**	0.799	[0.656; 0.882]	<0.001
	**FPCT-A *vs*. MR-A**	0.796	[0.652; 0.880]	<0.001
**Depth**	**FPCT-A *vs*. cast**	0.887	[0.808; 0.934]	<0.001
**(mm)**	**MR-A *vs*. cast**	0.866	[0.771; 0.921]	<0.001
	**FPCT-A *vs*. MR-A**	0.957	[0.927; 0.975]	<0.001

Intra-class correlation coefficients (ICC) for surface (in mm^2^) and depth (in mm) between flat panel computed tomography arthrography (FPCT-A), magnetic resonance arthrography (MR-A) and cast specimens. Data are presented with 95% confidence intervals (CI) and p-values

## Bland-Altman analysis

**Fig 2:**
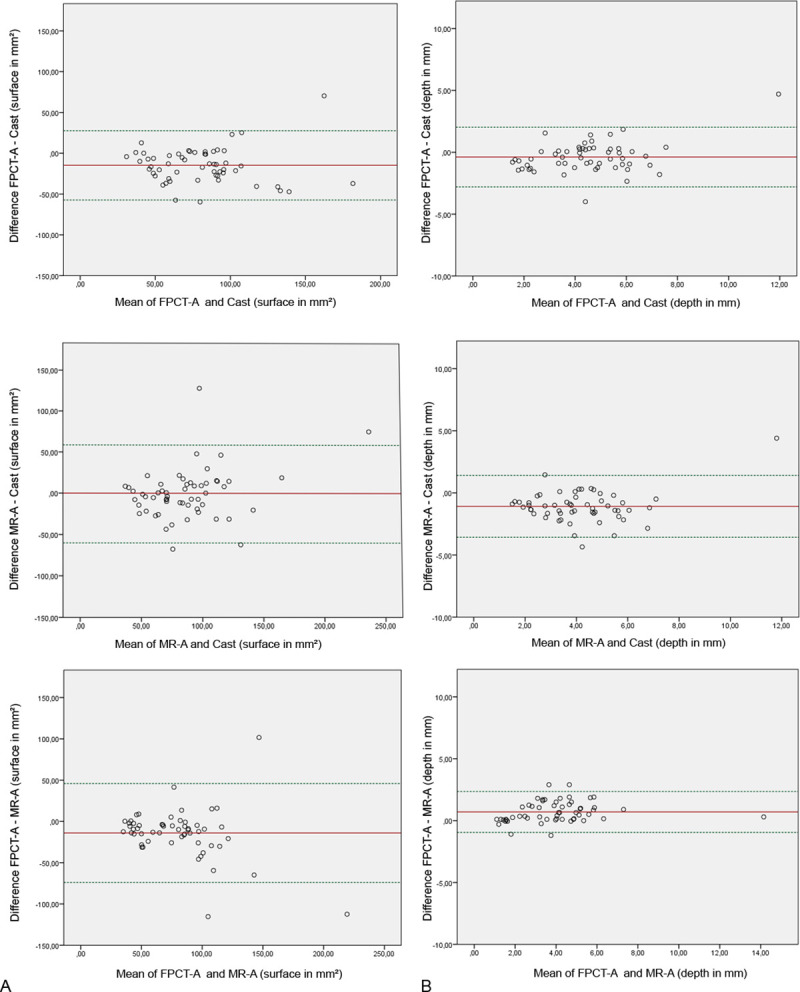
Bland-Altman plots. Bland-Altman plots for flat panel computed tomography arthrography (FPCT-A), magnetic resonance arthrography (MR-A) and cast specimens with mean differences of surface (A) and depth (B) measurements (red line) and ± 1.96*standard deviation (green dotted lines). The x-axis describes the mean of the data and the y-axis shows the difference of the data.

The Bland-Altman plots (Fig 2) demonstrate the mean differences of the compared imaging modalities and cast with almost all values within the limit of agreement range. There was no bias between FPCT-A, MR-A and cast.

**Fig 3:**
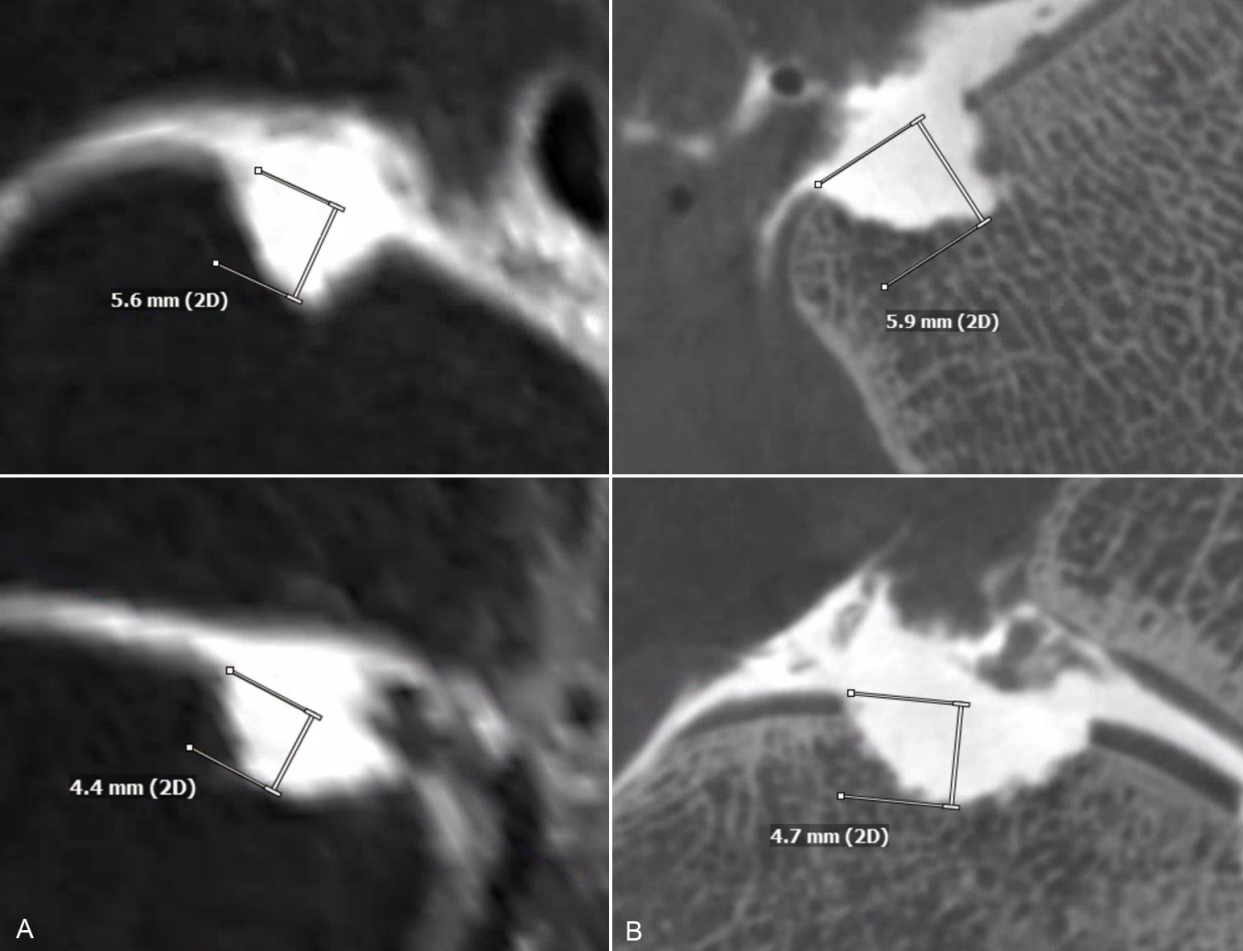
3D MR- and flat panel CT-arthrography measurements. Multiplane reconstruction of one single defect in the isotropic 3D space MR-sequence (A) with the corresponding planes in flat panel computed tomography arthrography (B) with marked depth measurements. The margins of the artificially created defect are clearly defined against the intra-articular contrast agent and the defect dimensions can be measured.

**Fig 4:**
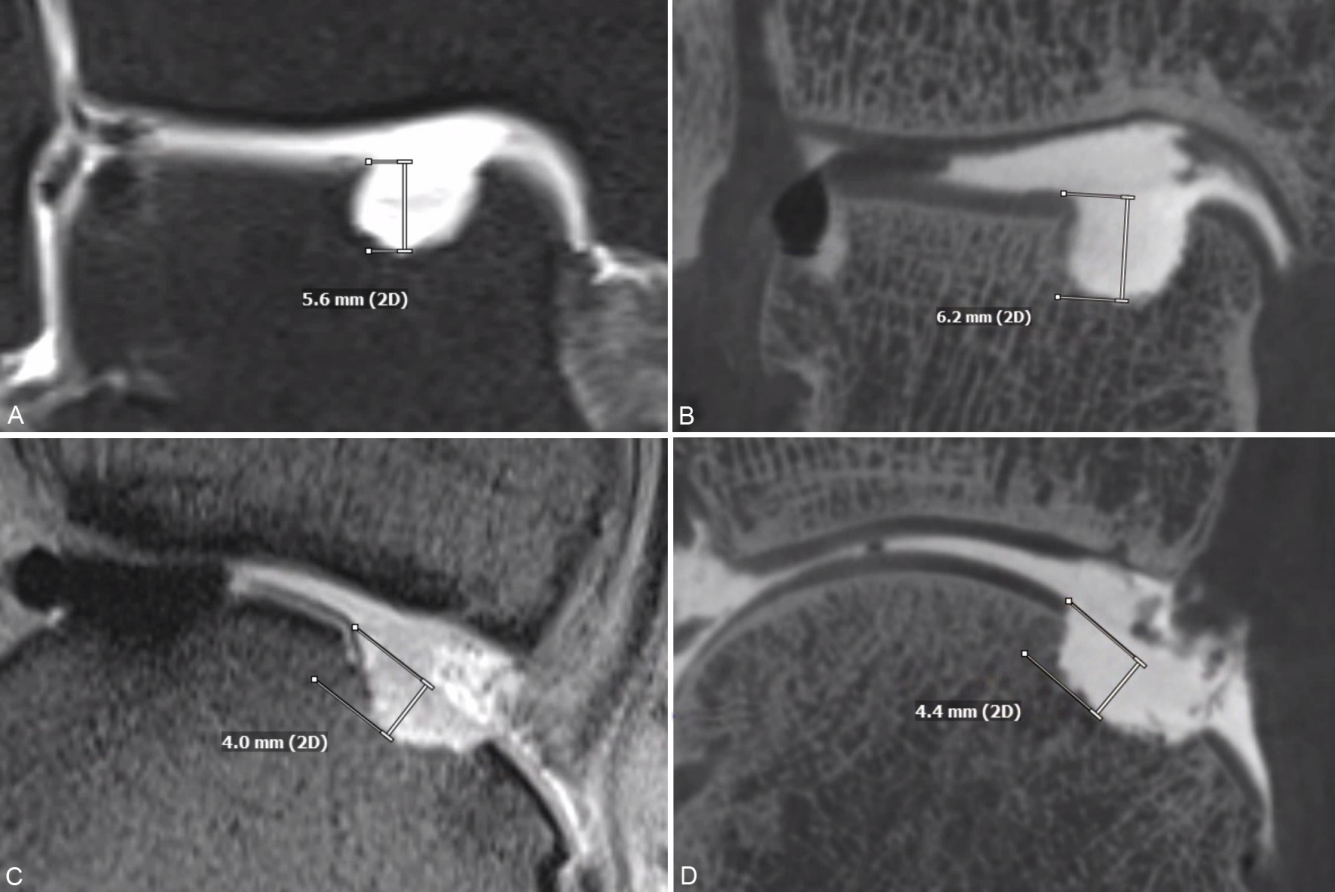
Different MR sequences and flat panel CT-arthrography measurements. Proton density weighted (PDw) coronal (A) and T1w sagittal (C) magnetic resonance arthrography (MR-A) sequences and the correspondingly reconstructed plane in FPCT-A images (B and D) of the same artificial defect. The marked depth measurements show larger values for the FPCT-A images.

**Fig 5:**
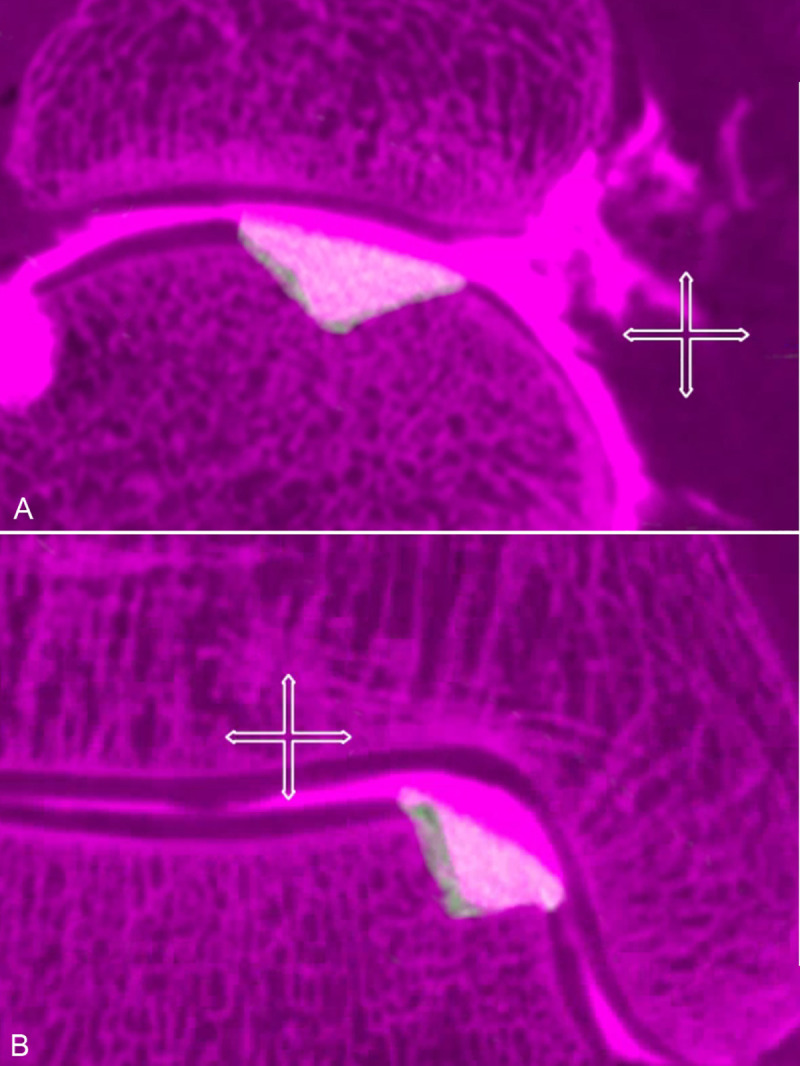
Fusion image of an osteochondral defect and the cast. Fusion mode images of an anteromedial defect in flat panel computed tomography arthrography (pink background) and the corresponding cast (brighter layer) in two different planes (A and B). Osteochondral defect and cast sizes can be directly compared. There is a visible overlay due to a slightly larger cast, explaining the mean differences of the measurements. The cross mark represents a 1 cm scale for both layers.

Image differences of spatial resolution between FPCT-A and MR-A are demonstrated in Fig 3 with a single defect reconstructed in two corresponding planes in MR-A (A) and FPCT-A (B) respectively. Fig 4 shows another single defect in the PD TSE FS (A) and T1 TSE (C) MR-sequences with the corresponding FPCT-A planes (B and D). Fig 5 represents a fusion image of an osteochondral talar defect in FPCT-A with an overlay FPCT-image of the corresponding cast specimen.

## Discussion

The main results of the current experimental study are: 1) The diagnostic capabilities are similarly excellent with both high resolution flat-panel computed tomography arthrography (FPCT-A) and magnetic resonance arthrography (MR-A). 100% of the lesions were detected with either technique. 2) Although there is very good cross-correlation between both techniques, FPCT-A was slightly more accurate than MR-A, which was most significant when measuring lesion depth. Those results are in line with previous studies proposing FPCT-A enables accurate evaluation of articular cartilage thickness and detection of cartilage defects [22–24]. This has even been demonstrated for smaller lesions compared to the defects in our study. Similarly, first *in vivo* results focusing on the wrist FPCT-A report an advantage over MR-A in regards to the visualization of the intrinsic ligaments and the cartilage [19].

### FPCT-A versus MR-A in the clinical context

For some time, MRI has been considered the standard procedure for the preoperative assessment of cartilage damage and localization to plan surgical access and the surgical type of procedure [27]. Recently, FPCT, a CT scanner model including volumetric imaging with ultra-high spatial resolution due to digital flat-panel detectors, was introduced [28]. This study suggests even a slightly superior performance of FPCT-A compared to MR-A regarding a precise osteochondral defect size measurement. We attribute the observed better correlations to FPCT´s higher spatial resolution of up to 0.11–0.12 mm in contrast to a 0.6 mm voxel size of the isotropic 3D MR sequence, the best possible resolution with the scanner. Thus, when interpreting the measurement differences, it is important to bear in mind the different resolutions associated with the different imaging modalities. An additional advantage of FPCT-A is the possibility of a free image plane adjustment. Although MR-A offers the advantage of completely avoiding any radiation exposure to the patient, The radiation dose is low for FPCT-A and comparable to a multi detector CT. Nevertheless, in the clinical setting, this needs consideration.

### Technical nuances and discrepancies

The defect length, width and depth were measured smaller in FPCT-A compared to the cast specimens. These differences were not very pronounced, as neither value did exceed 1.1 mm. Nonetheless, as the results were significant, we believe the differences are accounted for by technical limitations of the cast preparation. Especially defect surface measurements could have been somewhat overestimated for the cast measurements due to the narrow overhanging lines at the defect margins. Differences in the depth measurements could be additionally explained also by difficulties with the precise determination of the formal chondral layer, which is especially challenging at the talar edges. Some variation of the measurements between the different modalities can possibly be attributed to a range of artifacts caused by intra-articular air collections. This intra-articular air impaired the MR-A images to a greater extent than the FPCT-A measurements due to susceptibility artifacts.

An important result was FPCT-A measured lesion depth better (deeper) than MR-A. Vice versa, MR-A appeared to underestimate defect depth. This result is in line with previous studies showing a general trend of suboptimal defect size estimation by MR-A [29–31]. Campbell *et al*. demonstrated that preoperative knee MRIs underestimated the size of articular cartilage defects compared to arthroscopic results [30]. In some cases, MRI can even fail to detect osteochondral lesions altogether [32].

Still, there was overall good correlation between FPCT-A and MR-A measurements, as has been recently published in a recent study by Deng *et al*., [33]. Both, MRI and CT were reliable in the assessment of the size of subchondral cysts of talar defects and the MRI classification was well-correlated with the CT classification [33].

### Limitations

As the lesions evaluated in our study are artificial (surgically created), they do not fully resemble natural posttraumatic osteochondral lesions with chondral fissures, cartilage de-laminations or subtle subchondral defects. Accordingly, caution is needed when applying results of this study to the clinical practice.

As there were no ankles without osteochondral defects, there is no negative control group in this study. The radiologists knew to expect 4 lesions with every specimen. Thus, conclusions regarding sensitivity and specificity of the modalities are limited. Still, 100% of the lesions could be detected reliably with either method.

FPCT-A is a relatively new up and coming technique with only few reports discussing both *in vivo* and *ex vivo* defects [10, 34]. The cadaver specimens could be exposed to varying degrees of tissue de-composition, affecting the microstructure of tendons and cartilage. This could influence measurements using MR-A. Further, bone marrow edema is non-existent in the atraumatic cadaveric specimens, which would influence measurements *in vivo*.

A potential confounder may be the reference standard itself. Although the surface of the cast was essentially in line with the residual cartilage, the cast height (standard of the lesion depth) was measured systematically larger compared to both imaging modalities quantifying the lesion depth initially. This finding is reflected in the Bland-Altman plots. Nevertheless, this measurement approach is as close as possible to the real defect and cartilage thickness.

## Conclusions

FPCT-A and MR-A both demonstrate excellent diagnostic performance in detecting and quantifying osteochondral defects in cadaveric ankle joints (*ex vivo*). FPCT-A appears to quantify defect dimensions slightly better, specifically the defect depth.

## Abbreviations and acronyms

CI: confidence interval
FPCT-A: flat-panel computed tomography arthrography
FS: fat suppression
ICC: intraclass correlation coefficient
MDCT: multidetector computed tomography
MRI: magnetic resonance imaging
MR-A: magnetic resonance arthrography
PACS: picture archiving and communication system
PDw: proton density weighted
SD: standard deviation
SPACE: sampling perfection with application optimized contrasts using different flip angle evolution
T1w / T2w: T1 weighted / T2 weighted
TSE: turbo spin echo
